# Transcultural Adaptation of the Interpersonal Communication Competence Scale (ICCS) in Spanish Health Sciences Undergraduate Students

**DOI:** 10.3390/healthcare12242507

**Published:** 2024-12-11

**Authors:** David Sancho-Cantus, Pablo Álvarez-Nölting, Jesús Privado, Laura Cubero-Plazas, Marta Botella-Navas, Montserrat Cañabate-Ros

**Affiliations:** 1 Department of Nursing, Catholic University of Valencia, 46007 Valencia, Spain; david.sancho@ucv.es (D.S.-C.); pablo.alvarez@ucv.es (P.Á.-N.); laura.cubero@ucv.es (L.C.-P.); marta.botella@ucv.es (M.B.-N.); montse.canabate@ucv.es (M.C.-R.); 2 Doctoral Degree School, Catholic University San Vicente Mártir, 46001 Valencia, Spain; 3 Department of Methodology of Behavioral Sciences, Universidad Complutense de Madrid, Campus de Somosaguas, Pozuelo de Alarcón, 28223 Madrid, Spain; 4 Hospital Psychiatry Unit, Clinic Hospital of Valencia, 46010 Valencia, Spain

**Keywords:** ICCS, interpersonal communication, health sciences, validation, transcultural adaptation

## Abstract

**Background**: The communication skills of healthcare professionals directly impact patient interventions. Consequently, an improvement in healthcare quality indicators is observed. Objective: to adapt and validate the Interpersonal Communication Competence Scale (ICCS) in a Spanish university sample of health science students. **Methods**: A cross-sectional design was employed with a sample of 803 undergraduate students, utilizing the ICCS. **Results**: The structure and internal consistency of both the 30-item test and a short form version were analyzed. Additionally, factorial invariance, differential validity by gender, and the convergent and discriminant validity of the instrument were assessed. Benchmarks were also calculated. The complete scale, after removing five items, exhibited a total internal consistency of 0.721 and demonstrated a good fit to a hierarchical model with nine first-order factors and one second-order factor (GFI = 0.921, SRMR = 0.071). The 10-item short scale exhibited an internal consistency of 0.689 and fit a single-factor model (GFI = 0.977, SRMR = 0.059). Additionally, factorial invariance was established based on gender. Concerning convergent validity, the ICCS scales correlated with similar measures from the Social Skills Questionnaire and the Health Professional’s Communication Skills Scale. Regarding differential validity, significant differences were found only in self-disclosure, empathy, and immediacy, with higher scores in all three cases for women. **Conclusions**: The Spanish version of the ICCS demonstrates good psychometric properties and is a valid tool for assessing interpersonal communication competencies, both generally and specifically within health sciences. Additionally, the establishment of benchmarks in the ICCS will enable future evaluators to identify the position of their assessors relative to a university sample.

## 1. Introduction

### 1.1. Interpersonal Skills: Communication Competencies

Interpersonal skills, particularly communication between healthcare professionals and patients, play a pivotal role in determining the quality of care provided [1]. Effective communication has been shown to positively impact various aspects of healthcare, such as preventing professional burnout [2], improving quality indicators [3], enhancing health outcomes [4], reducing costs [5], and fostering treatment adherence [6]. For instance, robust communication strategies can reduce burnout episodes among professionals, enable earlier hospital discharge, minimize post-treatment complications, and improve adherence to prescribed treatments, ultimately resulting in cost reductions.

The effective use of interpersonal skills also fosters teamwork, enhances collaboration, and aids in conflict and negotiation management. Unresolved conflicts among healthcare professionals can adversely affect their work and compromise the quality of care and patient safety. As such, conflict resolution is a critical competency for future healthcare professionals [7]. Communication skills are fundamental to conflict resolution, as they enable listening, acceptance of differing viewpoints, and recognition of emotions that influence the communication process. These skills foster empathy, a key element in all social interactions [8].

Within the scope of communication skills, interpersonal communication (IC) refers to the exchange of information between individuals and includes elements such as openness, empathy, sociability, support, expressiveness, and immediacy. Empathy, in particular, significantly improves healthcare quality and encompasses cognitive (understanding others’ feelings), emotional (experiencing similar feelings), and behavioral (responding to those feelings) dimensions [9]. In clinical settings, IC is essential for patient care, facilitating practices such as fostering relationships, gathering and providing information, making decisions, responding to emotions, and addressing illness- and treatment-related behaviors [10]. Additional skills within IC include descriptiveness (the manner of giving and receiving information), ownership of feelings and thoughts, self-disclosure, behavioral flexibility, assertiveness, other-centeredness, and expressiveness [11].

The Relational Competence Model by Spitzberg provides a theoretical framework for approaching communicative competencies. This model identifies five core components of IC: motivation, knowledge, skills, context, and outcomes. Unlike earlier views that evaluated competence based solely on knowledge [12] or performance [13], this model emphasizes identifying behaviors likely to be considered competent, allowing room for improvement and training [14,15,16]. For this study, Rubin and Martin’s theoretical perspective on IC has been adopted, focusing on communication competencies in a general population [17]. Their approach provides a foundational understanding of IC and its role in fostering effective interactions in healthcare settings.

A chronological overview of the development of interpersonal and communication skills is presented in Table 1.

The specific objectives were as follows:

- Analyze the internal structure of the 30-item test to determine how many dimensions it comprises and whether they are hierarchically grouped with a general factor and other specific factors in which the different items are grouped.
- Verify if the internal structure of the 10-item test fits the presence of a general factor.
- Calculate the internal consistency of each of the factors obtained in the 30- and 10-item versions.
- Analyze the evidence of factorial invariance of the scale based on gender to see if the factorial structure is the same in both genders.
- Obtain evidence of convergent and discriminant validity by relating the ICCS to other measures: social and communication skills.
- Study the evidence of differential validity based on gender.
- Calculate the benchmarks for both tests for the evaluated sample.

### 1.2. Literature Review: Communication Assessment

In this first section, a review is conducted with the aim of describing the main instruments used in the evaluation of communication in general and in specific contexts.

There are various instruments that measure communication, and most of them do not analyze convergent validity or exhibit significant variability in reliability. Moreover, they differ considerably in terms of the theoretical models from which they emerge, their objectives, or the measurement methods employed by the authors to calculate the psychometric properties [6].

Among the various instruments designed to assess communication competencies, those included in Table 2 can be highlighted. There is a significant variability in the number of dimensions indicated by the authors in the questionnaires and scales that evaluate communication competencies, although those most frequently described are empathy, other-centeredness, and assertiveness.

In this second section, a detailed description of the instrument to be validated is provided, including information on previous validations and its respective psychometric properties across the different versions.

### 1.3. Interpersonal Communication Competence Scale

While there is a range of instruments available for assessing IC, they often do not evaluate all the relevant dimensions. To address this gap, the Interpersonal Communication Competence Scale (ICCS) has been developed. This tool offers insights into learning outcomes related to the enhancement of interpersonal communication skills and facilitates the assessment of progression in communication skill acquisition.

The original version of the questionnaire consisted of 60 items [17]. By removing items with lower reliability, the scale was reduced to 30 items, maintaining adequate internal consistency (α = 0.86). Regarding the different dimensions, reliability ranged from 0.26 (interaction management) to 0.55 (satisfaction with environmental or contextual control). It was validated in a sample of 309 high school students.

The principal component factorial analysis of the 30 items revealed that 25 items had their primary loadings on the first component extracted prior to rotation. According to McCroskey and Young [12], this suggests the likelihood of a single factor.

The 10 dimensions of the ICCS are [17] (1) self-disclosure, the ability to reveal aspects of one’s own personality to others; (2) empathy, a feeling of identification with others; (3) social relaxation, a lack of anxiety or apprehension in everyday social interactions; (4) assertiveness, defending one’s own rights without disregarding others’; (5) interaction management, the personal ability to manage ritual procedures in daily conversations; (6) other-centeredness (flexibility with others), a genuine interest in others, regarding both what they say and how they say it, paying special attention to both spoken and unspoken content; (7) expressiveness, the ability to communicate feelings through non-verbal behaviors (facial expressions and body gestures); (8) support, which in a communicative context, means being attentive to others and is descriptive (not evaluative), provisional (not definitive), spontaneous (not strategic), problem-solving oriented (not control of the situation), empathetic (not remote), and egalitarian (without superiority); (9) immediacy, being available for communication; and (10) environmental control, demonstrating one’s ability to tackle proposed objectives and meet needs.

There is also a brief version of the ICCS-SF [17] composed of the 10 items that showed the highest correlation with the total test. Its structure is unifactorial with low internal consistency (α = 0.63). Both versions, the 30-item and the 10-item, have a correlation of 0.96. These results are consistent with those reported by McCroskey and Young [12].

Regarding its adaptation to other populations, adaptations have been conducted in Denmark and Brazil. The Danish adaptation of the ICCS [26] was carried out with undergraduate nursing students and achieved high internal consistency (α = 0.77), with a 10-factor structure similar to the original version and a second-order general factor. The Brazilian adaptation of the scale [27] was condensed to 17 items, demonstrating high internal consistency (α = 0.82) and comprising five dimensions: environmental control, self-disclosure, assertiveness, interaction management, and immediacy.

### 1.4. Gender Differences in Communication Competence

Santos et al. [28] investigated communication competencies in students and identified significant differences related to variables such as age and gender. Their study, which involved a sample of 1079 nursing students, revealed that women tended to score higher in the immediacy dimension, while older participants achieved higher scores in the “environmental control” dimension.

Similarly, Misir et al. [29], in their study involving 585 university students, found a significant relationship between gender and interpersonal competence, with women scoring higher in the self-disclosure dimension.

In contrast, other studies, such as that of Park et al. [30], did not observe such differences. Nevertheless, concerning empathy, the existing literature generally supports the presence of significant gender differences, with women consistently demonstrating higher levels of this competency [31,32,33]. For instance, Öztürk and Kaçan [34] identified gender as a predictor of empathy skills in nursing students, with female participants achieving higher scores.

Studies conducted by Gutiérrez-Puertas et al. [9] have also reported significant differences in communication competencies related to gender and age, with males and older participants achieving higher scores [28].

The general objective was to adapt and validate the ICCS in a Spanish university sample of health science students. We focused on this group due to the importance of communication in the healthcare context [24].

Following the review, the importance of developing reliable and effective communication assessment instruments is emphasized. The advantages of using this questionnaire are as follows: good psychometric properties have been confirmed, reliability has been assessed across different versions of the questionnaire, benchmarks for interpretation have been provided, and gender differences have been studied. Additionally, this instrument has been validated in several languages and is widely used in research on interpersonal communication. Subsequent versions have been developed to refine its psychometric properties and ease of use.

Based on the above, the following research question was posed: What are the psychometric properties of the ICCS in its Spanish version?

## 2. Materials and Methods

This is a descriptive study for the validation of the psychometric properties of a questionnaire. The authors have used this term in the title to clarify that validation and adaptation to another language have been carried out.

### 2.1. Participants

The sample consisted of 803 participants, with a mean age of 21.16 years (SD = 4.72 years), of whom 77.8% were women, and all were students from the 1st to the 4th years of study. Their fields of study were as follows: 65.4% nursing, 5.7% psychology, 5.2% physiotherapy, 7.1% medicine, 5.4% nutrition, 3.0% podiatry, and the remaining percentage were from other university degrees. The selection of participants was intentional, and university students from the Faculty of Medicine and Health Sciences were recruited for this study. This faculty includes degrees such as medicine, nursing, nutrition, physiotherapy, and podiatry.

### 2.2. Instruments

- The Interpersonal Communication Competence Scale (ICCS) is a self-administered instrument that evaluates ten dimensions of interpersonal communication competence with 30 items (self-disclosure, empathy, social relaxation, assertiveness, other-centeredness, interaction management, expressiveness, support, immediacy, and environmental control), measured on a 5-point Likert scale [13].
- The Health Professionals’ Communication Skills Scale (CSS-HP) consists of 18 items, assessed with a 5-point Likert scale. It comprises four dimensions: informative communication, empathy, respect, and social skill. Table 2 shows the reliability of each of the test’s dimensions [25].
- The Social Skills Questionnaire (SSQ) is composed of 40 Likert-type items with 5 values, evaluating 10 skills: (1) interacting with people who attract me, (2) asserting one’s rights, (3) speaking in public/Interacting with authority figures, (4) keeping calm in embarrassing situations, (5) apologizing, (6) interacting with strangers, (7) expressing positive feelings, (8) facing situations where one might look foolish, (9) refusing requests, and (10) facing criticism. Table 2 displays the reliability indices of the test [35].

### 2.3. Procedure

The translation of the Interpersonal Communication Competence Scale into Spanish followed Brislin’s back-translation procedure. Initially, the whole scale was translated into Spanish by two independent bilingual experts, both of whom were specialized in English for health sciences. Then, two other bilingual translators, also with expertise in health sciences-specific English, independently translated the Spanish version back into English. Particular attention was given to the connotations and cultural nuances of words in both languages, ensuring the meaning of the original scale was preserved while considering the specific context of health sciences. Discrepancies between the original and back-translated versions were carefully analyzed, and any inconsistencies were resolved. Each sentence was reviewed for linguistic accuracy, and efforts were made to ensure the terminology would be understood by the target audience [36].

The questionnaires were administered in person, under the supervision of an evaluator, and were completed in a Google Forms survey. Prior to this, participants were provided with an informed consent form which they signed to participate in the study. They were informed that participation was voluntary and anonymous, and that they would not receive any financial compensation. Students received information about the study before completing the questionnaire. Approval was obtained from the Research Ethics Committee of the Catholic University of Valencia (UCV/2022-2023/033).

### 2.4. Statistical Analysis

First, the distribution of the different administered measures and their internal consistency indices were analyzed. Second, the factorial structure of the scale was studied through a confirmatory factor analysis. To assess the fit of the data, three types of goodness-of-fit indices were used:

Absolute fit indices, to see if the theoretical model fits the empirical data: the χ^2^/df index [37], where values below 3 indicate a good fit; the Goodness-of-Fit Index (GFI) [38], with values > 0.95 considered a good fit; and the Standardized Root Mean Square Residual (SRMR) [39], with values < 0.08 indicating a good fit [40].

Incremental fit indices, to compare the obtained model with the null model: the Normed Fit Index (NFI) [37], with values > 0.95 indicating a good fit.

Parsimony fit indices, which penalize the number of estimated parameters: the Parsimony Goodness-of-Fit Index (PGFI) [38] and the Parsimony Normed Fit Index (PNFI) [41], both with values > 0.50 indicating good fit.

The authors recommend 10 participants per indicator for factor analysis [42]. In our case, we have 802 participants and 30 indicators in the most complex model: 802/30 = 26.73 ≈ 27, which significantly exceeds the recommended minimum.

Third, the internal consistency of each scale of the test was calculated, as well as the correlation of each item with the total corrected test score to study the internal discrimination of the test. Fourth, the factorial structure of the brief scale proposed by Rubin and Martin [17] was verified, as well as an alternative based on the 10 items with the highest factorial weight in each of the obtained factors. Fifth, the internal consistency of the two obtained brief scales and their internal discrimination were calculated. Sixth, factorial invariance based on gender was checked for the scale. Seventh, Pearson correlations of the scale and its dimensions with the two additional scales and their dimensions were obtained to check the convergent and discriminant validity evidence of the ICCS. Eighth, the evidence of differential validity of the ICCS based on gender was studied using a Student’s *t*-test. Ninth, the benchmarks for the test were calculated: percentile scores, Z, standardized Z, and T scores.

The analyses were conducted using the statistical package SPSS V. 23 and the AMOS V. 23 software [43].

## 3. Results

*Descriptive Analysis*: In Table 3, the descriptive statistics for the different measures evaluated are presented. For all measures, the skewness is not greater than 2, and the kurtosis is not greater than 7 in absolute value, making the 12 items of the test suitable for using the maximum likelihood procedure [44]. Additionally, the internal consistency (Cronbach’s α) is reported for the social skills (SSQ) and communication (CSS-HP) measures. Overall, they are adequate as they have values of at least 0.70 [40], except for two subscales in both tests: facing ridicule (SSQ) and social skill (CSS-HP). Therefore, caution should be exercised when interpreting the results for these two subscales.

**Figure 1:**
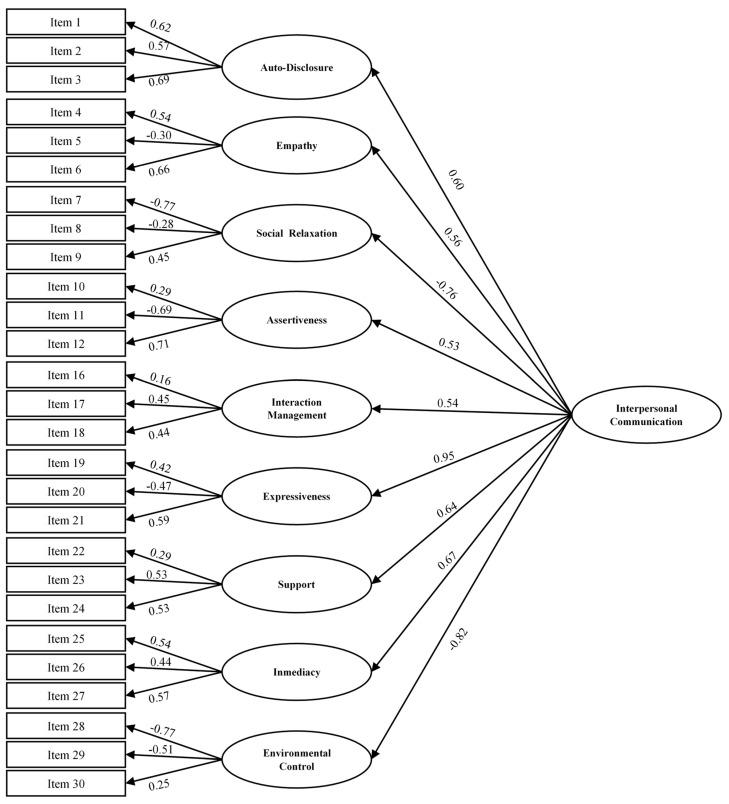
Hierarchical model for ICCS.

Internal Validity Evidence of the 30-Item Scale: A hierarchical model was estimated with a second-order general factor and 10 first-order factors for each dimension of the questionnaire. The model did not show multivariate normality using Bollen–Stine bootstrap [45] (*p* = 0.005), so the model was estimated using unweighted least squares, which do not require this type of normality. Table 3 shows the fit indices for the estimated model. The values indicate a moderate fit to the data. The model encountered issues in the altercentrism dimension; the factorial loading from the second-order factor onto this first-order factor was greater than 1. Additionally, the factorial loadings of the altercentrism indicators were below 0.40, the recommended minimum [40]. This suggested that it was advisable to remove this factor from the model and re-estimate the same model. Again, multivariate normality was not met, and the model was estimated using unweighted least squares. Figure 1 presents this model, and Table 4 shows the fit indices, which are slightly better than those of the previous model. As can be seen in Figure 1, most of the factorial loadings are above 0.40, and even the factorial loadings of the second-order factor on the first-order factors are greater than |±0.53|, reflecting a quite robust factorial structure.

Reliability Evidence of the 30-Item Scale: Internal consistency was calculated for the ICCS subscales and for the entire test using Cronbach’s α. Table 2 presents the results, which are acceptable for the total test (0.721), but not for the different first-order factors. One striking finding is a negative internal consistency for altercentrism (−0.209), confirming that this is a subscale that should be eliminated, as mentioned earlier. The internal discrimination analysis of each subscale was calculated with the correlation of each item in the scale with its total corrected. Most exceed the minimum value of 0.20, indicating that the items would be suitable [40]. However, three subscales show problems with internal discrimination: social relaxation, interaction management, and expressiveness. Nevertheless, none of the subscales achieve a minimum internal consistency of 0.70, making us cautious about the precision of these measures. Nonetheless, at a global level, the scale does present adequate internal consistency (0.721), and the different subscales contribute appropriately to the total test as their internal discrimination ranges from 0.35 to 0.54.

**Figure 2:**
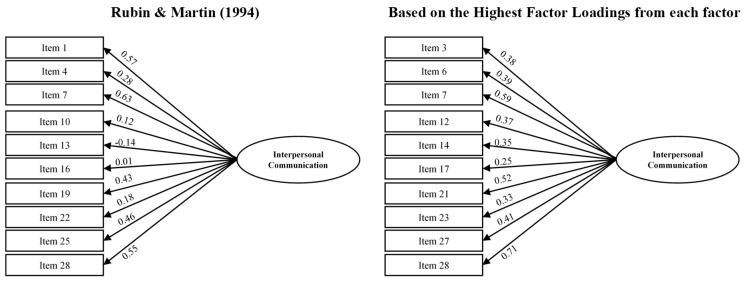
Factorial model for Rubin and Martin (1994) [17] and alternative based on the highest factor loadings from Model 1.

Internal Validity Evidence of the Short Scale: Following the original authors’ approach [17], a confirmatory model was calculated using the 10 items from the short version of the ICCS. Additionally, an alternative short version was proposed in which the items from each subscale with the highest factorial weight were used. In this case, we added the best item from altercentrism to this version to have a 10-item test as proposed by the authors. Both models did not show multivariate normality through Bollen–Stine bootstrapping [45] (*p* = 0.005), so they were estimated with unweighted least squares. Table 3 shows the fit indices, and Figure 2 displays the models with their factorial weights. As can be seen, the model based on the items proposed by the original authors shows a worse fit to the data than our model based on the highest factorial weights of each item relative to its subscale. Moreover, the factorial weights in our model are much higher (0.25 to 0.71) than those of Rubin and Martin’s model [17] (−0.14 to 0.63). For these reasons, we consider that our model better fits the data than that of the original scale authors.

Reliability Evidence of the Short Scale: We calculated the internal consistency of the test based on Rubin and Martin’s [17] items and our scale, obtaining an α = 0.454 for the original test and 0.689 for our approach. Additionally, the internal discrimination of the original test has four items (10, 13, 16, and 22) with item–total correlation values for the corrected subscale less than 0.20, whereas our short scale presents adequate values (0.20 to 0.54). Therefore, our short scale is suitable from both the factorial structure and reliability perspectives.

**Figure 3:**
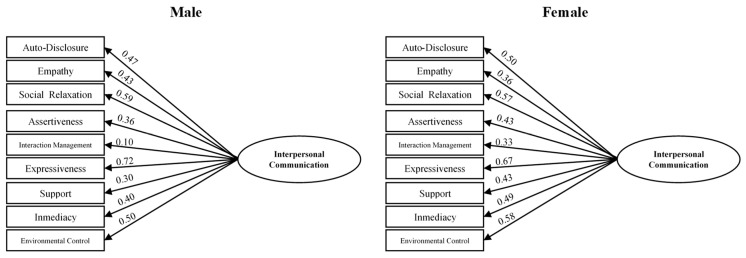
Hierarchical factor model for both genders.

Factorial Invariance Evidence by Gender: The analysis was conducted following Vanderberg and Lance’s approach [46], comparing different nested models to determine if they are equal across different independent groups: (1) an unrestricted model where factor loadings, covariance matrices, and error variances are different; (2) metric invariance of the models when the factor loadings are equal; and (3) strong invariance of the models when the covariance matrix is equal, and strict invariance when error variances are equal. Both models for both genders did not show multivariate normality using Bollen–Stine bootstrapping (*p* = 0.005), so they were estimated using unweighted least squares. Table 5 shows the fit goodness results obtained, and Figure 3 displays the two compared models. The Δχ^2^ increase was statistically significant (*p* < 0.001) for all comparisons, so we can assume that the factorial structure is different between both genders, with different values in their factor loadings.

Convergent and Discriminant Validity Evidence: Pearson correlations were calculated between the different ICCS subscales and the total score with the SSQ and CSS-HP. Table 6 presents the results obtained. In the italicized triangle, correlations between the different ICCS measures are shown, which are positive and, in most cases, have values of 0.30 or higher, reflecting convergence between the different measures, especially concerning the total measure. We chose the minimum of 0.30 as it is the medium effect size for correlation according to Cohen [21]. Regarding convergent validity (correlations in bold), correlations in bold in the gray box show how the different ICCS scales relate to similar measures from the SSQ and CSS-HP. Thus, for example, self-disclosure correlates with social skills (SSQ); empathy with empathy (SSQ); social relaxation with interacting with strangers (SSQ); expressiveness with public speaking (SSQ); support with positive feelings (SSQ); immediacy with positive feelings (SSQ); environmental control with social skills (SSQ); and total ICCS with almost all the subscales and totals of the SSQ and CSS-HP. These data clearly indicate that the ICCS shows adequate convergent validity with similar measures.

Evidence of Differential Validity: Mean differences were calculated using a Student’s *t*-test for the various ICCS subscales and the total score. The results obtained are presented in Table 7. Statistically significant differences were found only for self-disclosure, empathy, and immediacy, with higher scores in all three cases for women. Effect sizes were also calculated using Cohen’s d [21], where values of 0.20 are considered a small effect size, 0.50 a medium effect size, and 0.80 a large effect size. For the mean differences, medium to small effect sizes were found (0.30 to 0.40).

Norms: The norms for the evaluated scores are presented in Appendix B. For each direct score, percentile scores, standardized Z-scores (Z), normalized Z-scores, and T-scores (M = 50, SD = 10) were calculated. These data will allow future evaluators to determine the position of their subjects relative to a university sample. Separate norms were calculated for the subscales where gender differences were observed.

## 4. Discussion

In the current study, we present the validation of the ICCS scale in a sample of 803 university students majoring in health sciences. We opted to use this instrument because, despite not being specifically designed for healthcare professionals, it has been employed in this context in various studies with satisfactory results [28]. The internal structure of the scale appears to fit a hierarchical model with a general factor and nine second-order factors (GFI = 0.921, SRMR = 0.071). The altercentrism subscale was excluded from the model due to its poor performance in the evaluated sample. In the original scale by Martin and Rubin [17], there also appears to be low internal consistency, and they do not recommend dividing the scale into subscales. However, although the subscales exhibit limitations in reliability, the total score of the test in our study has adequate internal consistency (α = 0.721). Therefore, it should be used with caution at the subscale level due to its high measurement error. The subscales work well in estimating an overall factor that accounts for item variability, but on a practical or clinical level, we do not recommend using the subscale scores to make a diagnosis. We recommend using only the global scale scores because they have adequate internal consistency. Additionally, we tested a brief version of the scale consisting of 10 alternative items to those proposed by Rubin and Martin [17], which showed a good fit to a general factor (GFI = 0.977, SRMR = 0.059) considering the 10 items that load most on their respective subscales. This brief scale also has adequate internal consistency (α = 0.689). We found that the factorial structure of the scale is not the same in both genders, so this result should be considered when interpreting the scores. This finding had not been studied in previous validations of the questionnaire, which adds a novel aspect to the results of our research. Regarding different studies that have employed the ICCS, there are noticeable differences with our findings. Hald et al. [26] identified a general factor with six sub-dimensions, while in their Brazilian validation, Puggina et al. [27] described a total of five dimensions. Meanwhile, Mtsudaira et al. [47], in their adaptation of the ICCS to Japanese, described two general factors in the questionnaire, with five dimensions.

Regarding the evidence of convergent validity, the different subscales and the total score of the ICCS converge adequately with other similar measures (SSQ and CSS-HP). These findings can be explained because the CSS-HP and ICCS scales share common elements, such as empathy or assertiveness. Both can be used in similar contexts and evaluate aspects related to social interactions and communication [6]. Regarding the SSQ, there are different dimensions that, although not identical to those considered in the ICCS, do converge on some aspects related to social interaction, such as behaviors defending one’s own rights (assertiveness) and public speaking (which in the ICCS is considered “management of interaction”) [35].

Evidence of differential validity was found with higher scores for women in the subscales of self-disclosure, empathy, and immediacy. Except for empathy, the rest of the findings have not been supported by the literature, as there are no studies analyzing these variables in communication. While factorial invariance tries to contrast whether both sexes present different factorial weights in the different indicators (correlations between the factors and the items), mean differences seek to analyze whether the mean scores in the different scales are the same for both sexes. Generally, in view of an intervention, it is more useful to know if there are sex differences in the means in order to see in which sex more changes in the scale are expected after the intervention. In the study by Grice [22], for example, no significant sex differences were found in self-disclosure in a sample of 28 English high school teachers. Regarding empathy, Triffaux et al. [48] reported higher scores in both general empathy and its cognitive and affective dimensions in a sample of medical students. Other studies, such as that by Saha et al. [49], also point to significant differences in empathy with respect to gender, with women again scoring higher, in a sample of 790 health science students.

Our results align with those of previous studies, though they introduce the novel aspects of gender differentiation and the inclusion of specific benchmarks for interpreting the questionnaire.

Lastly, we present normative data for the individual subscales and the overall score of the ICCS to facilitate its utilization by subsequent researchers. Previous adaptations of this scale have not included normative data.

## 5. Conclusions

The primary limitations of the current study pertain to the characteristics of the sample evaluated, as it is exclusively composed of undergraduate students majoring in health sciences. Future research should aim to assess individuals from other academic disciplines and across different educational levels. Additionally, the sample consists predominantly of young individuals, and it would be valuable to investigate the scale’s outcomes in samples of varying age groups. Furthermore, the sample is skewed towards a higher percentage of females, which may have biased the results obtained. For example, it would be interesting to collect data from the social sciences, natural sciences, and engineering, where the number of men tends to be higher and the scale measurement could vary. In any case, it should be kept in mind that it is in the health sciences that interpersonal communication plays a key role in the therapist–patient relationship. For this reason, this type of population was chosen when validating the scale. It would also be insightful to examine the changes in the scale following an intervention aimed at enhancing communication competence, to assess evidence of scale sensitivity to change.

The ICCS demonstrates robust psychometric properties that make it suitable for use in assessing communication competencies, both at a general level and specifically within health sciences. This research contributes significant improvements over previous validations of the scale: it proposes a hierarchical structure from more to less generalized communication competencies, examines differential, convergent, and factorial invariance evidence by gender. Additionally, we provide norms for the test that could serve as a basis for its application in more applied clinical or educational settings.

The development of such questionnaires is relevant for both the evaluation and the subsequent development of communication skills, which are among the most important elements in the healthcare context between patients and healthcare professionals, as outlined throughout this research.

## Figures and Tables

**Table 1:** Conceptual evolution of interpersonal and communication skills. Source: own elaboration.

Period	Interpersonal Skills	Communication Skills
Before 1970	Initial approaches: viewed as general capacities for interaction. Social intelligence theories suggest they are key aspects of social adaptation.	Communication was primarily seen through rhetoric and information transmission; the process was unidirectional.
1970–1990	Social skills theories: psychology introduced training techniques to improve social and workplace interactions.	A bidirectional approach and interpersonal communication theory emerged: active listening, feedback, and context understanding.
1990–2010	Expanded to soft skills in the workplace, becoming essential in human resource management and leadership.	New technologies redefined communication. Virtual communication skills and the importance of non-verbal language were emphasized.
2010–…	Expanded to intercultural competencies and emotional intelligence, essential in a globalized world.	Adaptive and multicultural communication; digital media skills and competency in diverse contexts.

**Table 2:** Instruments assessing communicative competencies.

Instrument	Author/s	Objectives/Conclusions	Sample	Dimensions	Reliability	Validity
Communication Competence Scale	Wiemann (1977) [18]	To investigate the concept of communicative competence. The manner in which the interaction is managed contributes, in part at least, to fellow interactants’ perceptions of competence.	239 secondary education students	(1) General communicative competence. (2) Social relaxation.	α = 0.96 (0.33–0.92)	
Communication Self-Efficacy Questionnaire	Axnboe et al. (2016) [1]	To examine the content, internal structure, and relations with other variables of the new version of the self-efficacy questionnaire (SE-12). The questionnaire is useful for self-evaluation of clinical communication skills.	787 healthcare professionals (16.6% physicians, 69.2% nurses, 4.1% auxiliary staff) from Denmark, 82.6% women.	One factor.	α = 0.95; test–retest 0.64–0.84	
Conversational Skills Rating Scale	Spitzberg et al. (2009) [19]	To measure interpersonal skills in instructional contexts. CSRS appears to have great potential as an educational tool for use in assessing interpersonal competence.	707 university students	(1) Expressiveness. (2) Composure. (3) Altercentrism.	α = 0.89 (0.47–0.84)	Sex differences
Medical Communication Competence Scale	Cegala et al. (2009) [20]	To develop and partially assess a self-report scale for measuring doctors’ and patients’ perceptions of self-communication and other communication competence during a medical interview. The results provide support for the MCCS.	65 USA doctors (24.62% women) and 52 patients (65.38% women)	(1) Providing information. (2) Information-seeking and verification. (3) Socioemotional communication.	α = 0.75–0.82	
Communication Skills Checklist	Cohen et al. (1996) [21]	To compare the reliability of a checklist and rating scales. Standardized patients’ ratings may be more efficient than SP checklists.	178 USA medical students		α = 0.65 and 0.76	
Patient-Centered Communication Tools (PaCT)	Grice et al. (2017) [22]	To develop a comprehensive instrument specific to student pharmacist–patient communication skills, and to determine face, content, construct, concurrent, and predictive validity and reliability of the instrument. PaCT is a useful instrument for assessing student pharmacist communication skills with patients.	216 USA pharmacy students	23 aspects related to clinical communication.	Test–retest = 0.75	
American Board of Internal Medicine’s (ABIM) Patient Assessment Survey	Symons et al. (2009) [23]	To evaluate residents’ self-assessment of their communication skills and professionalism in dealing with patients, and to evaluate the psychometric properties of a self-assessment questionnaire. This appears to be an internally consistent and reliable tool for residents’ self-assessment of communication skills and professionalism.	130 USA doctors (39.23% women)	(1) Development of interpersonal relationships with patients. (2) Information provided to the patients.	α = 0.86 (0.36–0.79)	
Health Professionals’ Skills and Self-Efficacy	Lasitter et al. (2023) [24]	To develop and validate an instrument to measure health professionals’ communication skills and self-efficacy specifically related to working with individuals with disabilities. The final 18-item inventory that demonstrates strong validity and reliability.	37 USA healthcare and physical education professionals (51% women)	(1) Skills. (2) Self-efficacy.	α = 0.91–0.94	
Health Professionals’ Communication Skills Scale	Leal-Costa et al. (2015) [25]	Creation of a communication skills scale in health professionals, CSS-HP. Experts positively appreciated the constructs defined and the items created from those definitions, which were modified and eliminated according to these results.	927 healthcare professionals: physicians (21.3%), nurses (48.5%), and nursing assistants (30.2%); 70.33% women. Mean age: 40.95 (SD+/− 10.069).	(1) Empathy. (2) Informative communication. (3) Respect. (4) Assertiveness.	α = 0.88	
Interpersonal Communication Competence Scale	Rubin and Martin (1994) [17]	Development of a self-report Interpersonal Communication Competence Scale. Previously developed ICC instruments are too limited in scope or too difficult to complete to be entirely useful in instructional settings.	247 university students (57.6% women)	(1) Self-disclosure. (2) Empathy. (3) Social relaxation. (4) Assertiveness. (5) Altercentrism. (6) Interaction management. (7) Expressiveness. (8) Support. (9) Immediacy. (10) Environmental control.	α = 0.41–0.77	Convergence with cognitive flexibility (r = 0.49) and communicative flexibility (r = 0.40)
Interpersonal Communication Competence Scale (Spanish Version)	Sancho et al. (2024)	To adapt and validate the Interpersonal Communication Competence Scale in a Spanish university sample of health science students. The Spanish version of the ICCS demonstrates good psychometric properties and is a valid tool for assessing interpersonal communication competencies, both generally and specifically within health sciences.	803 undergraduate students	(1) Self-disclosure. (2) Empathy. (3) Social relaxation. (4) Assertiveness. (5) Altercentrism. (6) Interaction management. (7) Expressiveness. (8) Support. (9) Immediacy. (10) Environmental control.	α = 0.721	

**Table 3:** Descriptive statistics, internal consistency (Cronbach’s α), and internal discrimination (corrected item–total correlation) for the measures.

Measures	M	SD	Skewness	Kurtosis	α	R_Item–Total correlation_
Self-Disclosure (ICCS)	10.34	2.37	−0.17	−0.38	0.648	0.40 to 0.50
Empathy (ICCS)	11.97	1.76	−0.32	0.19	0.445	0.26 to 0.32
Social Relaxation (ICCS)	10.98	1.98	−0.02	−0.41	0.429	0.12 to 0.39
Assertiveness (ICCS)	10.74	2.33	−0.21	−0.45	0.550	0.26 to 0.44
Altercentrism (ICCS)	11.05	1.54	0.20	0.05	−0.209	−0.18 to 0.01
Interaction Management (ICCS)	9.61	1.93	−0.29	0.31	0,319	0.11 to 0.24
Expressiveness (ICCS)	10.54	2.25	−0.34	0.16	0.460	0.12 to 0.42
Support (ICCS)	11.87	1.92	−0.50	−0.01	0.442	0.23 to 0.35
Immediacy (ICCS)	12.75	1.86	−0.63	−0.15	0.505	0.29 to 0.37
Environmental Control (ICCS)	10.39	2.01	−0.14	0.50	0,531	0.29 to 0.42
Total ICCS	110.25	10.38	−0.11	−0.29	0.721	0.35 to 0.54
Brief ICCS	38.24	3.85	−0.28	−0.17	0.689	0.20 to 0.54
Interacting with Strangers (SSQ)	13.38	3.55	−0.14	−0.42	0.798	
Positive Feelings (SSQ)	17.42	2.92	−1.25	1.29	0.819	
Facing Criticism (SSQ)	15.85	3.04	−0.43	−0.26	0.828	
Interacting with Acquaintances (SSQ)	11.65	4.72	0.03	−0.94	0.923	
Calmness in the Face of Criticism (SSQ)	14.28	3.10	−0.36	0.21	0.733	
Public Speaking/Interacting with Superiors (SSQ)	13.27	3.89	−0.17	−0.62	0.826	
Facing Ridicule (SSQ)	12.14	3.40	−0.12	−0.09	0.636	
Asserting Rights (SSQ)	14.75	3.42	−0.47	−0.02	0.766	
Apologizing (SSQ)	17.86	2.33	−1.10	0.94	0.771	
Rejecting Requests (SSQ)	14.88	3.38	−0.43	−0.25	0.785	
Total Social Skills (SSQ)	145.50	20.36	−0.14	0.06	0.807	
Empathy (CSS-HP)	23.07	2.47	−1.76	4.38	0.843	
Informative Communication (CSS-HP)	26.59	2.92	−0.92	0.42	0.715	
Respect (CSS-HP)	14.10	1.40	−1.86	3.92	0.764	
Social Skill (CSS-HP)	15.23	2.50	−0.10	−0.42	0.545	
Total Communication Skills (CSS-HP)	83.57	7.95	−1.10	1.53	0.877	

**Table 4:** Goodness-of-fit indices for the models.

Model	χ^2^/df	GFI	NFI	PGFI	PNFI	SRMR
ICCS, 30 items including altercentrism	25.11	0.908	0.764	0.771	0.764	0.074
ICCS, 25 items without altercentrism	21.22	0.921	0.795	0.768	0.714	0.071
ICCS, 10 items (Rubin & Martin (1994) [17])	6.10	0.977	0.804	0.617	0.625	0.067
ICCS, 10 items	5.28	0.977	0.912	0.622	0.709	0.059

Note. χ^2^/df = chi square/degrees of freedom; GFI = Goodness-of-Fit Index; NFI = Normed Fit Index; PGFI = Parsimony Goodness-of-Fit Index; PNFI = Parsimony Normed Fit Index; SRMR = Standardized Root Mean Square Residual.

**Table 5:** Factorial invariance by gender.

Specified Model	χ^2^	gl	χ^2^/gl	GFI	NFI	SRMR
Model A. Without restrictions (unconstrained)	3272.47	36	90.91	0.967	0.900	0.090
Model B. Equal factorial weights (structural weights)	3617.91	28	129.21	0.963	0.889	0.099
Model C. Equal factorial weights and variance–covariance matrix (structural covariances)	3767.85	27	139.55	0.962	0.885	0.103
Model D. Equal factorial weights, variance–covariance matrix, and error variance (measurement residuals)	3886.04	18	215.89	0.961	0.881	0.100
**Model Comparison**	**Δχ^2^**	**Δgl**	**p**			
Models A and B (metric invariance)	445.44	8	<0.001			
Models B and C (strong metric invariance)	149.94	1	<0.001			
Models C and D (strict metric invariance)	118.19	9	<0.001			

**Table 6:** Pearson correlations between ICCS and CHASO, and EHC-PS.

Measures	1	2	3	4	5	6	7	8	9
Self-Disclosure (ICCS)	*1.00*								
2. Empathy (ICCS)	*0.14*	*1.00*							
3. Social Relaxation (ICCS)	***0.30***	*0.19*	*1.00*						
4. Interaction Management (ICCS)	*0.12*	*0.20*	*0.08*	*1.00*					
5. Expressiveness (ICCS)	***0.42***	*0.21*	***0.38***	*0.14*	*1.00*				
6. Support (ICCS)	*0.22*	***0.31***	*0.26*	*0.10*	*0.25*	*1.00*			
7. Immediacy (ICCS)	***0.30***	*0.28*	*0.29*	*0.10*	*0.20*	***0.39***	*1.00*		
8. Environmental Control (ICCS)	*0.19*	*0.20*	***0.32***	*0.23*	***0.38***	*0.12*	*0.27*	*1.00*	
9. Total ICCS	***0.58***	***0.49***	***0.61***	***0.42***	***0.68***	***0.51***	***0.57***	***0.62***	*1.00*
10. Interacting with Strangers (SSQ)	0.25	0.07	**0.50**	0.19	0.23	0.15	0.28	0.29	**0.43**
11. Positive Feelings (SSQ)	0.23	0.20	0.24	0.02	0.21	**0.36**	**0.42**	0.17	**0.38**
12. Facing Criticism (SSQ)	0.18	0.15	0.19	0.24	0.28	0.13	0.20	**0.30**	**0.43**
13. Interacting with Acquaintances (SSQ)	0.20	0.02	0.25	0.13	0.15	0.13	0.18	0.18	0.28
14. Calmness in the Face of Criticism (SSQ)	0.08	0.13	0.25	0.13	0.11	0.21	0.18	0.18	0.27
15. Public Speaking/Interacting with Superiors (SSQ)	0.18	0.08	**0.39**	0.17	**0.34**	0.17	0.21	**0.35**	**0.43**
16. Facing Ridicule (SSQ)	0.24	0.01	0.17	0.23	0.10	0.07	0.20	0.15	0.28
17. Asserting Rights (SSQ)	0.14	0.01	0.21	0.19	0.18	0.04	0.17	0.26	**0.32**
18. Apologizing (SSQ)	0.21	0.23	0.14	0.02	0.19	**0.32**	**0.31**	0.13	**0.31**
19. Rejecting Requests (SSQ)	0.14	0.00	0.16	0.13	0.13	0.08	0.16	0.22	0.28
20. Total Social Skills (SSQ)	**0.31**	0.13	**0.43**	0.25	**0.32**	0.26	**0.37**	**0.38**	**0.57**
21. Empathy (CSS-HP)	0.19	**0.31**	0.15	0.07	0.14	**0.30**	**0.38**	0.17	**0.35**
22. Informative Communication (CSS-HP)	0.14	**0.34**	0.21	0.04	0.21	0.28	**0.35**	0.22	**0.39**
23. Respect (CSS-HP)	0.09	0.20	0.13	0.01	0.11	0.24	**0.32**	0.14	0.26
24. Social Skill (CSS-HP)	0.16	0.18	0.16	0.15	0.19	0.19	0.24	0.29	**0.35**
25. Total Communication Skills (CSS-HP)	0.19	**0.33**	0.21	0.09	0.21	**0.32**	**0.40**	0.26	**0.43**

Note: Correlations ≥ |±0.07| are statistically significant at the 5% level. Correlations ≥ 0.30 are bolded.

**Table 7:** Descriptives, mean differences, and effect sizes for the ICCS.

Measure	Male M (SD)	Female M (SD)	*t*-Test	Cohen ’s d
Self-Disclosure	9.79 (2.36)	10.49 (2.35)	*t_801_* = −3.54, *p* < 0.001	−0.30
Empathy	11.46 (1.74)	12.12 (1.73)	*t_801_* = −4.52, *p* < 0.001	−0.38
Social Relaxation	11.10 (2.06)	10.95 (1.96)	*t_801_* = 0.87, *p* = 0.384	0.07
Assertiveness	10.96 (2.32)	10.68 (2.33)	*t_801_* = 1.42, *p* = 0.157	0.12
Interaction Management	9.85 (1.88)	9.54 (1.94)	*t_801_* = 1.89, *p* = 0.059	0.16
Expressiveness	10.39 (2.30)	10.59 (2.23)	*t_801_* = −1.05, *p* = 0.293	−0.09
Support	11.72 (2.02)	11.91 (1.89)	*t_801_* = −1.15, *p* = 0.251	−0.10
Immediacy	12.17 (1.81)	12.91 (1.84)	*t_801_* = −4.76, *p* < 0.001	−0.40
Environmental Control	10.50 (1.80)	10.36 (2.07)	*t_801_* = 0.80, *p* = 0.425	0.07
ICCS, 25 items	97.93 (9,70)	99.56 (10.43)	*t_801_* = −1.87, *p* = 0.062	−0.15
ICCS, 10 items	37.80 (3.64)	38.36 (3.90)	*t_801_* = −1.72, *p* = 0.086	−0.16

Note. M = mean; SD = standard deviation.

## Data Availability

The data presented in this study are available on request from the corresponding author.

## Appendix A. Interpersonal Communication Competence Scale

1. I allow my friends to see who I really am.
2. Others know what I am thinking.
3. I reveal to others how I feel.
4. I can put myself in other people’s shoes.
5. I do not know exactly what others are feeling.
6. Others think I understand them.
7. I feel comfortable in social situations.
8. I feel relaxed in meetings with small groups of people.
9. I feel insecure when I am with a group of strangers.
10. When I have been offended, I confront the person who has offended me.
11. I find it difficult to defend my ideas.
12. I know how to defend my rights.
13. My conversations are quite one-sided.
14. I let others know that I understand what they are saying.
15. My mind wanders during conversations.
16. My conversations are characterized by smoothly switching from one topic to another.
17. I take control of conversations by negotiating the topics discussed.
18. In conversations with friends, I notice not only what they say, but also what they don’t say.
19. My friends know when I am happy or sad.
20. It is difficult to find the right words to express myself.
21. I express myself well verbally.
22. My communication tends to be descriptive, not evaluative.
23. I communicate with others as an equal.
24. I am described by others as a caring person.
25. My friends genuinely believe that I care about them.
26. I try to look others in the eye when I talk to them.
27. When I feel close to someone I tell them.
28. I achieve my communication goals.
29. I am able to convince others to adopt my position.
30. I find it difficult to convince others to do what I want them to do.

## Appendix B. Percentiles, Z-Scores, Normalized Z-Scores, and T-Scores for the ICCS

**Centile**	**Z_n_**	**T**	**Auto-Disclosure Total**	**Z** **Auto-Disclosure Total**	**Auto-Disclosure Male**	**Z** **Auto-Disclosure Male**	**Auto-Disclosure Female**	**Z** **Auto-Disclosure Female**	**Empathy Total**	**Z** **Empathy Total**	**Empathy** **Male**	**Z** **Empathy Male**	**Empathy** **Female**	**Z** **Empathy Female**	**Social** **Relaxation**	**Z** **Social Relaxation**	**Assertiveness**	**Z** **Assertiveness**		
1	−2.33	26.70	4.00	−2.68	4.00	−2.45	5.00	−2.34	8.00	−2.26	6.79	−2.68	8.00	−2.38	7.00	−2.01	5.00	−2.46		
5	−1.64	33.60	6.00	−1.83	6.00	−1.61	7.00	−1.49	9.00	−1.69	9.00	−1.41	9.00	−1.80	8.00	−1.51	7.00	−1.61		
10	−1.28	37.20	7.00	−1.41	7.00	−1.18	7.00	−1.49	10.00	−1.12	9.00	−1.41	10.00	−1.23	8.00	−1.51	8.00	−1.18		
15	−1.04	39.60	8.00	−0.99	7.00	−1.18	8.00	−1.06	10.00	−1.12	10.00	−0.84	10.00	−1.23	9.00	−1.00	8.00	−1.18		
20	−0.84	41.60	8.00	−0.99	8.00	−0.76	8.00	−1.06	10.80	−0.66	10.00	−0.84	11.00	−0.65	9.00	−1.00	9.00	−0.75		
25	−0.67	43.30	9.00	−0.57	8.00	−0.76	9.00	−0.63	11.00	−0.55	10.00	−0.84	11.00	−0.65	10.00	−0.49	9.00	−0.75		
30	−0.52	44.80	9.00	−0.57	9.00	−0.33	9.00	−0.63	11.00	−0.55	11.00	−0.26	11.00	−0.65	10.00	−0.49	10.00	−0.32		
35	−0.39	46.10	9.00	−0.57	9.00	−0.33	10.00	−0.21	11.00	−0.55	11.00	−0.26	11.00	−0.65	10.00	−0.49	10.00	−0.32		
40	−0.25	47.50	10.00	−0.14	9.00	−0.33	10.00	−0.21	11.00	−0.55	11.00	−0.26	12.00	−0.07	11.00	0.01	10.00	−0.32		
45	−0.13	48.70	10.00	−0.14	9.00	−0.33	10.00	−0.21	12.00	0.02	11.00	−0.26	12.00	−0.07	11.00	0.01	11.00	0.11		
50	0.00	50.00	10.00	−0.14	10.00	0.09	11.00	0.22	12.00	0.02	11.00	−0.26	12.00	−0.07	11.00	0.01	11.00	0.11		
55	0.13	51.30	11.00	0.28	10.00	0.09	11.00	0.22	12.00	0.02	11.00	−0.26	12.00	−0.07	11.00	0.01	11.00	0.11		
60	0.25	52.50	11.00	0.28	10.00	0.09	11.00	0.22	12.00	0.02	12.00	0.31	13.00	0.51	12.00	0.52	11.00	0.11		
65	0.39	53.90	11.00	0.28	11.00	0.51	11.00	0.22	13.00	0.59	12.00	0.31	13.00	0.51	12.00	0.52	12.00	0.54		
70	0.52	55.20	12.00	0.70	11.00	0.51	12.00	0.64	13.00	0.59	12.00	0.31	13.00	0.51	12.00	0.52	12.00	0.54		
75	0.67	56.70	12.00	0.70	12.00	0.94	12.00	0.64	13.00	0.59	13.00	0.89	13.00	0.51	12.00	0.52	12.00	0.54		
80	0.84	58.40	12.00	0.70	12.00	0.94	13.00	1.07	14.00	1.15	13.00	0.89	14.00	1.09	13.00	1.02	13.00	0.97		
85	1.04	60.40	13.00	1.12	12.00	0.94	13.00	1.07	14.00	1.15	13.00	0.89	14.00	1.09	13.00	1.02	13.00	0.97		
90	1.28	62.80	13.00	1.12	13.00	1.36	14.00	1.49	14.00	1.15	14.00	1.46	14.00	1.09	14.00	1.53	14.00	1.40		
95	1.64	66.40	14.00	1.54	14.00	1.78	14.00	1.49	15.00	1.72	15.00	2.03	15.00	1.66	14.00	1.53	14.00	1.40		
99	2.33	73.30	15.00	1.97	14.21	1.87	15.00	1.92	15.00	1.72	15.00	2.03	15.00	1.66	15.00	2.03	15.00	1.83		
N			803	803	178	178	625	625	803	803	178	178	625	625	803	803	803	803		
Mean	0.00	50.00	10.34	0.00	9.79	0.00	10.49	0.00	11.97	0.00	11.46	0.00	12.12	0.00	10.98	0.00	10.74	0.00		
SD	1.00	10.00	2.37	1.00	2.36	1.00	2.35	1.00	1.76	1.00	1.74	1.00	1.73	1.00	1.98	1.00	2.33	1.00		
**Centile**	**Z_n_**	**T**	**Interaction Management**	**Z** **Interaction Management**	**Expressiveness**	**Z** **Expressiveness**	**Support**	**Z** **Support**	**Immediacy** **Total**	**Z** **Immediacy Total**	**Immediacy Male**	**Z** **Immediacy Male**	**Immediacy Female**	**Z** **Immediacy Female**	**Environ-Mental Control**	**Z** **Environ-Mental Control**	**ICCS**	**Z** **ICCS**	**Brief ICCS**	**Z** **Brief ICCS**
1	−2.33	26.70	4.04	−2.89	5.00	−2.46	7.00	−2.54	8.00	−2.55	8.00	−2.30	8.00	−2.67	5.00	−0.17	75.00	−2.35	29.00	−2.40
5	−1.64	33.60	6.00	−1.87	7.00	−1.57	9.00	−1.49	9.00	−2.02	9.00	−1.75	9.00	−2.13	7.00	1.83	81.00	−1.77	31.00	−1.88
10	−1.28	37.20	7.00	−1.35	8.00	−1.13	9.00	−1.49	10.00	−1.48	10.00	−1.20	10.00	−1.58	8.00	2.83	85.00	−1.38	33.00	−1.36
15	−1.04	39.60	8.00	−0.83	8.00	−1.13	10.00	−0.97	11.00	−0.94	10.00	−1.20	11.00	−1.04	9.00	3.83	88.00	−1.09	34.00	−1.10
20	−0.84	41.60	8.00	−0.83	9.00	−0.68	10.00	−0.97	11.00	−0.94	11.00	−0.65	11.00	−1.04	9.00	3.83	90.00	−0.89	35.00	−0.84
25	−0.67	43.30	8.00	−0.83	9.00	−0.68	11.00	−0.45	12.00	−0.40	11.00	−0.65	12.00	−0.49	9.00	3.83	93.00	−0.60	36.00	−0.58
30	−0.52	44.80	9.00	−0.32	10.00	−0.24	11.00	−0.45	12.00	−0.40	11.00	−0.65	12.00	−0.49	9.00	3.83	94.00	−0.51	36.00	−0.58
35	−0.39	46.10	9.00	−0.32	10.00	−0.24	11.00	−0.45	12.00	−0.40	11.00	−0.65	12.00	−0.49	10.00	4.83	96.00	−0.31	37.00	−0.32
40	−0.25	47.50	9.00	−0.32	10.00	−0.24	12.00	0.07	12.00	−0.40	12.00	−0.09	13.00	0.05	10.00	4.83	97.00	−0.21	37.00	−0.32
45	−0.13	48.70	9.00	−0.32	10.00	−0.24	12.00	0.07	13.00	0.13	12.00	−0.09	13.00	0.05	10.00	4.83	99.00	−0.02	38.00	−0.06
50	0.00	50.00	10.00	0.20	11.00	0.20	12.00	0.07	13.00	0.13	12.00	−0.09	13.00	0.05	10.00	4.83	100.00	0.08	39.00	0.20
55	0.13	51.30	10.00	0.20	11.00	0.20	12.00	0.07	13.00	0.13	13.00	0.46	13.00	0.05	11.00	5.83	101.00	0.17	39.00	0.20
60	0.25	52.50	10.00	0.20	11.00	0.20	13.00	0.59	13.00	0.13	13.00	0.46	14.00	0.59	11.00	5.83	102.00	0.27	39.00	0.20
65	0.39	53.90	10.00	0.20	11.00	0.20	13.00	0.59	14.00	0.67	13.00	0.46	14.00	0.59	11.00	5.83	103.00	0.37	40.00	0.46
70	0.52	55.20	11.00	0.72	12.00	0.65	13.00	0.59	14.00	0.67	13.00	0.46	14.00	0.59	11.00	5.83	105.00	0.56	40.00	0.46
75	0.67	56.70	11.00	0.72	12.00	0.65	13.00	0.59	14.00	0.67	13.25	0.60	15.00	1.14	12.00	6.83	106.00	0.66	41.00	0.72
80	0.84	58.40	11.00	0.72	12.00	0.65	14.00	1.11	15.00	1.21	14.00	1.01	15.00	1.14	12.00	6.83	108.00	0.86	42.00	0.98
85	1.04	60.40	12.00	1.24	13.00	1.09	14.00	1.11	15.00	1.21	14.00	1.01	15.00	1.14	12.00	6.83	110.00	1.05	42.00	0.98
90	1.28	62.80	12.00	1.24	13.00	1.09	14.00	1.11	15.00	1.21	15.00	1.56	15.00	1.14	13.00	7.83	112.00	1.24	43.00	1.24
95	1.64	66.40	13.00	1.76	14.00	1.54	15.00	1.63	15.00	1.21	15.00	1.56	15.00	1.14	14.00	8.83	115.80	1.61	44.00	1.50
99	2.33	73.30	14.00	2.27	15.00	1.98	15.00	1.63	15.00	1.21	15.00	1.56	15.00	1.14	15.00	9.83	122.00	2.22	46.96	2.26
N			803	803	803	803	803	803	803	803	178	178	625	625	803	803	803	803	803	803
Mean	0.00	50.00	9.61	0.00	10.54	0.00	11.87	0.00	12.75	0.00	12.17	0.00	12.91	0.00	10.39	0.00	99.2	0.00	38.24	0.00
SD	1.00	10.00	1.93	1.00	2.25	1.00	1.92	1.00	1.86	1.00	1.81	1.00	1.84	1.00	2.01	1.00	10.29	1.00	3.85	1.00

## References

[1] Axboe M., Christensen K., Kofoed P., Ammentorp J. (2016). Development and validation of a self-efficacy questionnaire (SE-12) measuring the clinical communication skills of health care professionals. BMC Med. Educ..

[2] Cebrià J., Palma C., Segura J., Gracia R., Pérez J. (2006). El entrenamiento en habilidades de comunicación podría ser un factor preventivo del síndrome de burnout en médicos de familia. Rev. Psiquiatr. Fac. Med. Barc..

[3] Marino F., Alby F., Zucchermaglio C., Fatigante M. (2023). Digital technology in medical visits: A critical review of its impact on doctor-patient communication. Front. Psychiatr..

[4] Chandra S., Mohammadnezhad M. (2021). Doctor-Patient Communication in Primary Health Care: A Mixed-Method Study in Fiji. Int. J. Environ. Res. Public Health.

[5] Andersson S.O., Barde A., André M., Kristiansson P. (2020). Consultation skills of final year medical students in Sweden: Video-recorded real-patient consultations in primary health care assessed by Calgary-Cambridge Global Consultation Rating Scale, a pilot study. Assoc. Med. Educ. Eur..

[6] Leal-Costa C., Tirado-González S., Van-der Hofstadt Román C.J., Rodríguez-Marín J. (2016). Creation of the Communication Skills Scale in Health Professionals, CSS-HP. An. Psicol..

[7] Brown T., Yu M., Etherington J. (2021). Listening and interpersonal communication skills as predictors of resilience in occupational therapy students: A cross-sectional study. Br. J. Occup. Ther..

[8] Sexton M., Orchard C. (2016). Understanding healthcare professionals’ self-efficacy to resolve interprofessional conflict. J. Interprof. Care.

[9] Gutiérrez-Puertas L., Márquez-Hernández V., Gutiérrez-Puertas V., Granados-Gámez G., Aguilera-Manrique G. (2020). Interpersonal communication, empathy, and stress perceived by nursing students who use social networks. J. Adv. Nurs..

[10] King A., Hoppe R.B. (2013). “Best practice” for patient-centered communication: A narrative review. J. Grad. Med. Educ..

[11] Bochner A., Kelly C. (1974). Interpersonal competence: Rationale, philosophy, and implementation of a conceptual framework. Speech Teach..

[12] McCroskey J., Young T. (2006). The Use and Abuse of Factor Analysis in Communication Research. Hum. Communic. Res..

[13] Rubin R. (1982). Assessing speaking and listening competence at the college level: The communication competency assessment instrument. Commun. Educ..

[14] Bateman A., Braithwaite B., Bromley D., Evans J., Garlick S., Gibb R., Japp J., Rhodes G., Murphy L., McKinlay B. (2010). The Brain Injury Handbook.

[15] Koskinen S. (1998). Quality of life 10 years after a very severe traumatic brain injury (TBI): The perspective of the injured and the closest relative. Brain Inj..

[16] Thomsen I. (1974). The patient with severe head injury and his family. A follow-up study of 50 patients. Scand. J. Rehab Med..

[17] Rubin R., Martin M. (1994). Development of a measure of interpersonal communication competence. Commun. Res. Rep..

[18] Wiemann J.M. (1977). Explication and test of a model of communicative competence. Hum. Commun. Res..

[19] Spitzberg B., Hurt H. (1987). The measurement of interpersonal skills in instructional contexts. Commun. Educ..

[20] Cegala D., Coleman M., Turner W. (1998). The development and partial assessment of the medical communication competence scale. Health Commun..

[21] Cohen J. (1992). A Power Primer. Psychol. Bull..

[22] Grice G., Gattas N., Prosser T., Voorhees M., Kebodeaux C., Tiemeier A., Berry T., Wilson A., Mann J., Juang P. (2017). Design and Validation of Patient-Centered Communication Tools (PaCT) to Measure Students’ Communication Skills. Am. J. Pharmac. Educ..

[23] Symons A.B., Swanson A., McGuigan D., Orrange S., Akl E.A. (2009). A tool for self-assessment of communication skills and professionalism in residents. BMC Med. Educ..

[24] Lassiter J., Campbell A., Taliaferro A., Zimmerman S. (2023). Measuring Health Professionals’ Skills and Self-Efficacy for Communicating with Individuals with Disabilities: Instrument Development and Validation. J. Health Comm..

[25] Leal-Costa C., Tirado González S., Ramos-Morcillo A., Ruzafa-Martínez M., Díaz Agea J., van-der Hofstadt Román C. (2020). Communication Skills and Professional Practice: Does It Increase Self-Efficacy in Nurses?. Front. Psychol..

[26] Hald S., Baker F., Ridder H. (2015). A preliminary psychometric evaluation of the interpersonal communication competence scale for aquired brain injury. Brain Inj..

[27] Puggina A., Silva M. (2014). Interpersonal Communication Competence Scale: Brazilian translation, validation and cultural adaptation. Acta Paul. Enferm..

[28] Santos J., Copelli F., Balsanelli A., Sarat C., Menegaz J., Trotte L., Stipp M., Soder R. (2019). Interpersonal communication competence among nursing students. Rev. Lat.-Am. Enferm..

[29] Mısır S., Demir A., Koydemir S. (2020). The relationship between perceived interpersonal competence and self-disclosure in an online context: The moderating role of shyness. Int. J. Psychol..

[30] Park J., Caltabiano N., Hajhashemi K. (2019). The Role of User Demographics, Self-Efficacy and Interpersonal Competence on Communication Style Preferences of Rural University Students. Austr Int. J. Rural. Educ..

[31] Altwalbeh D., Khamaiseh A., Algaralleh A. (2018). Self-reported empathy among nursing students at a University in Jordan. Open Nurs. J..

[32] Berduzco-Torres N., Medina P., San-Martín M., Delgado Bolton R., Vivanco L. (2021). Non-academic factors influencing the development of empathy in undergraduate nursing students: A cross-sectional study. BMC Nurs..

[33] Ertuğ N. (2018). The investigation of levels of empathy in nurse candidates. Bezmialem. Sci..

[34] Öztürk A., Kaçan H. (2022). Compassionate communication levels of nursing students: Predictive role of empathic skills and nursing communication course. Perspect. Psychiatr. Care.

[35] Caballo V., Salazar I., Rivera-Riquelme M., Piqueras J. (2017). Development and validation of a new social skills assessment instrument: The Social Skills Questionnaire. Beh Psychol..

[36] Brislin R. (1970). Back-translation for cross-cultural research. J. Cross Cult. Psychol..

[37] Bentler P.M., Bonett D.G. (1980). Significance tests and goodness of fit in the analysis of covariance structures. Acad. Psychol. Bull..

[38] Jöreskog K.G., Sörbom D. (1993). LISREL 8: User’s Guide.

[39] Hu L., Bentler P.M. (1999). Cutoff criteria for fi t indexes in covariance structure analysis: Conventional criteria versus new alternatives. Struct. Equ. Model. Multidiscip. J..

[40] Hair J.F., Anderson R.E., Tatham R.L., Black W.C. (1999). Análisis Multivariante.

[41] James L.R., Mulaik S.A., Brett J.M. (1982). Causal Analysis: Models, Assumptions and Data.

[42] Byrne B.M. (2001). Structural Equation Modeling with AMOS Basic Concepts, Applications, and Programming.

[43] Arbuckle J.L. (2006). Amos 7.0 User’s Guide.

[44] West S.G., Finch J.F., Curran P.J., Hoyle R.H. (1995). Structural equation models with non-normal variables. Structural Equation Modeling: Concepts, Issues and Applications.

[45] Bollen K.A., Stine R.A., Bollen K.A., Long J.S. (1993). Bootstrapping goodness-of-fit measures in structural equation models. Testing Structural Equation Models.

[46] Vanderberg R., Lance C. (2000). A review and synthesis of the measurement invariance literature: Suggestions, practices, and recommendations for organization research. Organ. Res. Methods.

[47] Matsudaira T., Fukuhara T., Kitamura T. (2008). Factor structure of the Japanese Interpersonal Competence Scale. Psychiatry Clin. Neurosci..

[48] Triffaux J.M., Tisseron S., Nasello J. (2023). Medical students’ empathy during the COVID-19 pandemic: A cross-sectional study. Int. J. Emot. Educ..

[49] Saha S., Narang R., Aggarwal V., Brinda G., Kavita D. (2021). Comparison of Self-Reported Empathy Levels among Dental Undergraduate Students in Northern India: A Questionnaire-Based Cross-Sectional Study. Contemp. Clin. Dent..

